# Developing loneliness interventions for people with personality disorders: A call to action

**DOI:** 10.1111/nyas.15367

**Published:** 2025-05-14

**Authors:** Matthias A. Reinhard, Frank Padberg

**Affiliations:** ^1^ Department of Psychiatry and Psychotherapy LMU University Hospital Munich Germany; ^2^ DZPG (German Center for Mental Health) Partner Site Munich‐Augsburg Munich Germany

**Keywords:** adverse childhood experiences, loneliness, personality disorder, personality functioning

## Abstract

Loneliness is common in personality disorders (PDs) and may contribute to their severity and persistence. Systematic research on loneliness interventions for PD is sparse but clearly needed. According to the Alternative Model of Personality Disorders, loneliness may be related to personality functioning and may link to domains of self (identity and self‐direction) and interpersonal functioning (empathy and intimacy). A deeper understanding of the interaction between perceived loneliness and individual dysfunction across these domains is essential for establishing novel psychological interventions that effectively treat loneliness. As a first step in developing a valid therapy model, we suggest a modular framework that encompasses loneliness‐associated perceptions, cognitions, feelings, and behaviors that can be targeted with specific strategies. To further develop this framework, additional empirical research, for example, clinical studies on loneliness‐tailored interventions in PD, should be performed in parallel with more conceptual research that challenges and integrates current hypotheses and theories.

## Graphical abstract

Loneliness may contribute to the severity and persistence of personality disorders (PDs) and therefore represents a clinically important condition and a target for specific psychological interventions. Mechanisms maintaining loneliness in PD could be addressed with specific techniques for different levels of impaired self‐ and interpersonal functioning (e.g., dysfunctional core beliefs, impaired emotion regulation, and difficulties in developing and maintaining close and mutual relationships).

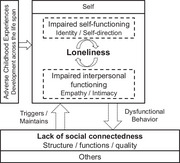

## INTRODUCTION

Loneliness is an aversive experience and describes a distressing emotional state that results from the perceived mismatch of desired and actual social relationships.^1^ More than six decades ago, Reichmann^2^ characterized the phenomenon of loneliness and the attitude of clinicians and scientists toward it as follows: “Loneliness seems to be such a painful, frightening experience that people will do practically everything to avoid it. This avoidance seems to include a strange reluctance on the part of psychiatrists to seek scientific clarification of the subject. Thus, it comes about that loneliness is one of the least satisfactorily conceptualized psychological phenomena, not even mentioned in most psychiatric textbooks.”

For people with personality disorder (PD), feeling disconnected and lonely represents a particularly pervasive experience, as was reported by qualitative^3^ and systematic reviews.^4^, ^5^ Compared with people with other mental health conditions, such as psychosis, depression, and anxiety disorders, on average people with PD report the highest levels of loneliness.^6^ Furthermore, loneliness may contribute to the severity and persistence of PD^3^, ^5^ across different types of the disorder, such as borderline PD^7^, ^8^, ^9^ and narcissistic personality.^10^, ^11^, ^12^ Loneliness is associated with impaired personality functioning, suggesting that PD and loneliness are closely intertwined.^13^

Numerous theoretical models describe PD in terms of self‐concepts and interpersonal functioning. The psychodynamic concept of personality functioning includes a set of basic abilities and capacities to regulate oneself and one's relationships that encompass the sense of self, interpersonal contact, and one's model of relationships (i.e., domains that are typically impaired in both people with PD and lonely individuals).^13^ In this context, loneliness has been related to identity diffusion (i.e., deficits in developing a cohesive and integrated sense of self and others), which is shaped by early interactions with significant others.^12^ In addition, previous research has shown that loneliness and PD share common factors and mechanisms that contribute to their development and maintenance and that encompass intrapersonal factors, such as social hypervigilance, information processing biases, and rejection expectations, and common interpersonal factors, such as social withdrawal.^5^

Both loneliness and PD are hypothesized to stem from adverse childhood experiences (ACEs),^5^ which—according to ICD‐11—constitute a recognized risk factor for the development of PD.^14^ From a developmental perspective, ACEs, which include various forms of neglect and abuse, may strongly interfere with the formation of healthy personality functioning and thus lead to impaired self‐ and interpersonal functioning—the core of PD.^13^ In addition, ACEs were found to be associated with loneliness later in life.^5^, ^15^, ^16^ This relationship may be mediated by various pathways, including insecure attachment style,^12^, ^17^ difficulties in emotion regulation,^18^ increased rejection sensitivity,^8^ and deleterious object representations^19^ (i.e., by pathways that may parallel those between ACE and PD).

Because loneliness has a major influence on physical and mental health^20^, effective and evidence‐based interventions are urgently needed.^21^ In a recent review, Hickin et al.^21^ concluded that psychological interventions effectively reduce loneliness with a small to medium effect size. The authors evaluated a considerable range of interventions and found a large heterogeneity in terms of study quality and effectiveness,^21^ suggesting that specific interventions may need to better target an individual's needs.^22^ Regarding the various types of interventions, the authors observed that reminiscence interventions, social identity approaches, and cognitive‐behavioral therapy were the most effective.^21^ Similarly, a meta‐analysis by Masi et al.^23^ compared four different types of loneliness interventions, namely, improving social skills, enhancing social support, increasing opportunities for social interaction, and addressing maladaptive social cognitions, and found that the last was the most effective.

A recent evidence and gap map of in‐person loneliness interventions gives an overview of methods that are provided on various delivery levels, including self‐guided, interpersonal, community‐based, or societal delivery.^24^ Psychotherapeutic interventions are typically delivered as a self‐guided approach or on an interpersonal level and include interventions that change cognition, improve social skills, or enhance social support. The authors suggested four mechanisms of change that could guide interventions: enhancing social connections, maintaining existing connections, creating new connections, or changing negative social cognitions. All four of these mechanisms may be of particular relevance in PD because the disorder is characterized by impaired social connections and negative cognitions about oneself and others.

Loneliness interventions that target social cognitions (e.g., information processing biases toward negative content) appear to be promising because these cognitions are closely associated with loneliness.^25^ For example, information‐processing biases include hypervigilance for social threat, hostile attribution styles, and negative evaluations of oneself and others, especially in ambiguous situations.^25^ Furthermore, lonely individuals expect to be rejected by others, and this expectation and its behavioral consequences (e.g., hostile reactions, avoidance, or withdrawal) may serve as factors that maintain or aggravate isolation and loneliness.^8^ A recent intervention study showed that interpretation styles can be effectively targeted with cognitive bias modification training.^26^ The training, which comprised a single session, significantly reduced social threat, hostile interpretations, and loneliness. To further increase their effectiveness, loneliness interventions may focus on loneliness‐associated emotions, perceptions, and behavior. For instance, Käll et al.^27^ highlighted various cognitive‐behavioral intervention targets, such as the emotional responses that accompany loneliness (e.g., anxiety), lack of social skills, and loneliness‐reinforcing behaviors. Thus, the authors concluded that effective interventions should address both intrapersonal (the negative self‐concept) and interpersonal factors (social avoidance).

Although loneliness is a pervasive, burdensome, and clinically relevant experience in PD, it may be overlooked by therapists, and specific psychological interventions are urgently needed. Moreover, loneliness interventions have not been developed specifically for PD, even though certain features of the disorder may necessitate the development of specific loneliness interventions. Therefore, in this article, we propose a holistic working model of loneliness in PD and suggest potential interventions that warrant further investigation.

## LONELINESS AND THE ALTERNATIVE MODEL OF PERSONALITY DISORDERS

A PD is defined as a disorder with enduring and maladaptive patterns of inner experience, cognition, and behavior that lead to distress and impaired functioning in various facets of social areas.^14^, ^28^ In the DSM‐5 Alternative Model of Personality Disorders (AMPD), criterion A characterizes a PD by impairment in the two higher‐order dimensions, that is, self (identity, self‐direction) and interpersonal functioning (empathy, intimacy).^28^ This criterion was developed from and parallels the evolution of the psychodynamic concepts of personality functioning. Criterion A is further accompanied by maladaptive traits that specifically describe personality styles (i.e., the criterion B domains of negative affectivity, detachment, psychoticism, antagonism, and disinhibition). ICD‐11 follows the dimensional conceptualization of DSM‐5 but adapts it slightly (e.g., ICD‐11 uses the alternative formulation of the trait anankastia instead of psychoticism).^14^ Recent studies have found associations of AMPD criteria A and B with loneliness, which may inspire the development of loneliness interventions. These studies are briefly described below.

### Loneliness and AMPD criterion A

Regarding associations with AMPD criterion A, Kunz et al.^29^ reported that in a sample of psychiatric inpatients, loneliness was correlated with self‐ and observer‐rated measures (i.e., with the Level of Personality Functioning Scale [LPFS] Brief Form 2.0^30^—a short version of the original LPFS—and the Semi‐Structured Interview for Personality Functioning DSM‐5, StiP‐5.1^31^) of self‐ and interpersonal dysfunction. The strongest association with loneliness was found for impaired self‐direction and impaired intimacy. Similarly, in a larger representative population sample, Labonté and Kealy^32^ observed an association between loneliness and self‐ and interpersonal dysfunction. Furthermore, by using the 12‐item self‐report version of the Operationalized Psychodynamic Diagnosis Structure Questionnaire^33^ to assess participants’ level of personality functioning, Ernst et al.^13^ found a strong association of loneliness with personality functioning. In the study by Ernst et al.^13^, personality functioning explained even more variance of loneliness scores than other known and established risk factors, such as living alone or having no partner. Finally, to identify domains of personality dysfunction (i.e., identity, self‐direction, empathy, and intimacy) that may particularly account for loneliness, Finch and Kealy^11^ investigated the association between loneliness and narcissistic vulnerability with the Self and Interpersonal Functioning Scale.^34^ Intimacy functioning, but not empathy, identity, or self‐direction, was found to significantly mediate the relationship between narcissistic vulnerability and loneliness.

In sum, recent studies from various theoretical backgrounds have investigated the association between loneliness and personality functioning with a range of methods for assessing personality functioning. A common conclusion of these studies could be that loneliness may be related to individual domains of AMPD criterion A, as further described in the following.

Identity comprises aspects such as an integer self‐concept, adequate self‐esteem, and the ability to self‐regulate one's feelings and behaviors.^28^ People with PD may have a negative self‐image and core beliefs that they are not worthy to be loved.^32^ Interpersonal difficulties and a lack of social connections may be interpreted as individual deficits, and this interpretation—together with associated self‐blame and feelings of shame and guilt—may additionally confirm such core beliefs. Consequently, people with PD may feel inferior to others and have reduced self‐worth; these feelings may be additionally fostered by internalized stigma and increase the feeling of loneliness.^6^ People with PD may also feel overly superior (i.e., have increased self‐worth and devalue others), which may provoke rejection by others and similarly increase the risk of being alone and lonely. In addition, people with PD may have difficulties experiencing a sense of connectedness and belonging, which could be rooted in early adversities (e.g., ACE such as emotional neglect or abuse). As a result, similar to lonely individuals without PD, people with PD may have negative expectations toward others (e.g., they may expect to be rejected).^8^ Furthermore, their ability to self‐regulate loneliness may be impaired because they have difficulties in recognizing and regulating their own emotions.^29^ As a result, loneliness may be felt as being more intense, longer lasting, and more aversive, depending on the accompanying emotions, which may vary between individuals and include shame, guilt, grief, and anger. Thus, people with PD may have an impaired ability to self‐regulate and self‐soothe and even believe that they have not earned self‐compassion. Finally, unstable and unpredictable behaviors may result from identity‐related characteristics of people with PD.^32^

Self‐direction describes a person's ability to self‐reflect, pursue individual goals, and adhere to prosocial norms.^28^ Therefore, people with PD may have difficulties deciding with whom they want relationships and which kind of relationships they want and also in adequately directing and controlling their own interpersonal behaviors.^32^ PD may rather be associated with avoidance goals (because they try to protect themselves against further rejection) than with prosocial and reconnecting goals. Research has shown that avoidance goals are more generally observed in people who are also experiencing loneliness.^25^

Empathy is based on cognitive and emotional theory‐of‐mind functions and includes the capacity to understand others, mutual perspective‐taking, and knowing one's impact on others.^28^ In a recent review, Hayward et al.^35^ reported the relevance of empathy as an innate dysfunction in people with borderline PD that may account for other clinical features in this condition such as emotional instability and interpersonal distress. The authors emphasized the role of cognitive and emotional empathy as a core deficit in borderline PD and discussed empathy training interventions. More generally, people with PD may have difficulties adequately seeing and interpreting others’ emotions and behaviors in situations where social cues are important, and they may even be hypervigilant toward putative signals of being rejected. In turn, this negatively biased perception may reduce their ability to recognize offers from others to join and reconnect. Furthermore, interpersonal relationships may suffer from a lack of mutual perspective‐taking. Individuals with PD may have an impaired ability to realize and anticipate how others are affected by their behavior (e.g., hostile‐aggressive or overly intrusive behavior), which in turn may provoke rejection and increase isolation and loneliness.

Intimacy describes the ability to develop and maintain close and mutually satisfying relationships.^28^ People with PD may lack the social skills to adequately start new relationships or uphold them (e.g., the social skills needed to manage and repair conflicts). In addition, people with PD may even have the core belief that closeness is aversive and dangerous. PD may be accompanied by hostile attitudes toward others and fear of being too exploitable. The resulting missing and unstable social relationships may increase the feeling of reduced belonging and connectedness in PD.

### Loneliness and AMPD criterion B

The AMPD criterion B domains comprise specific maladaptive personality traits and also show significant correlations with loneliness. Whereas Romero and Alonso^36^ found significant associations of loneliness with negative affectivity, detachment (e.g., withdrawal and intimacy avoidance), and psychoticism in a college sample, Roche et al.^37^ observed a correlation of loneliness only with negative affectivity and detachment but not psychoticism, antagonism, or disinhibition in an adolescent sample. Finally, in a sample of older twins, Freilich et al.^38^ found significant correlations between loneliness and all five traits, although they observed the strongest correlations for negative affectivity and detachment. All criterion B personality traits, including anankastia as defined by ICD‐11, may interplay with feelings of loneliness.

Negative affectivity describes the tendency of people with PD to experience negative emotions, such as anxiety, hostility, and emotional lability.^28^ Feelings of shame, guilt, anxiety, and anger, which are associated with loneliness, are common in individuals with high negative affectivity. In addition, negative emotions may mutually reinforce themselves. Therefore, increased negative affectivity in PD may also increase the tendency to experience loneliness.

Detachment encompasses tendencies to maintain social and emotional distance (e.g., via social withdrawal, avoiding intimacy, suspiciousness, and anhedonia).^28^ Social detachment and withdrawal may lead to a smaller social network, which constitutes a risk factor for loneliness. Emotional detachment (e.g., avoiding intimacy) may be associated with a reduced quality of a social network. Taken together, several dimensions of increased detachment may contribute to and maintain feelings of loneliness.

Psychoticism encompasses eccentric beliefs, experiences, and behaviors that deviate from the general population.^28^ It usually leads to misinterpretation and rejection by others. Furthermore, it may be associated with a feeling of not being understood by others and accompanied by social withdrawal, which may increase feelings of loneliness.^39^

Antagonism describes the tendency to be manipulative, deceitful, and callous and to have a desire for grandiosity.^28^ Individuals with high antagonism levels, such as vulnerable, narcissistic individuals, were found to perceive others more negatively and less kindly and to show more jealousy, dissatisfaction, and aggression within relationships (for an overview, see Ref. 11). In addition, they may feel that they are superior to others and that others are not good enough for closer relationships. Consequently, antagonistic individuals may keep away from or reject others. At the same time, people may socially withdraw from antagonistic individuals or reject them because of their hostile behavior, which can also increase disconnection and feelings of loneliness.

Disinhibition encompasses increased distractibility and irresponsible and impulsive behavior.^28^ Impulsivity may provoke and hurt social relationships and thus reduce the quality and quantity of social relationships and increase loneliness.

Anankastia as defined by ICD‐11 describes the tendency to have rigid standards of perfection and rigid control over situations,^14^ a trait that may lead to increased isolation. Invalid assumptions about the self and others (e.g., “things work better when I do them on my own”) may be provocative and lead to further rejection. Furthermore, the excessive control of one's emotional expression may hinder the development of close and intimate relationships.

## LONELINESS INTERVENTIONS FOR PD

To date, no psychological intervention has shown effective for specifically treating loneliness in PD. However, targeting AMPD criteria A and B with psychotherapeutic interventions can be assumed to have a positive impact on loneliness because of the associations and putative mechanisms outlined above. Vice versa, specifically addressing loneliness may alleviate the severity of PD. So far, no specific treatment protocol exists for criterion A,^40^ but treatments that address criterion A appear to be promising for alleviating loneliness. For instance, Marčinko and Sutara^12^ suggested addressing identity by helping to develop a more accurate and positive self‐image and stable self‐worth (instead of shame or constant doubt).

Regarding criterion B, Barlow et al.^41^ proposed a treatment protocol for negative affectivity, the so‐called Unified Protocol (UP). The UP was developed as a transdiagnostic intervention for emotional disorders^42^ and targets negative affectivity on different levels associated with emotional dysregulation. For instance, it consists of different modules that focus on mindful awareness of emotions (perception), cognitive flexibility (cognition), and changing emotional action tendencies and actions (behavior). Furthermore, participants are exposed to emotions (feelings) and interoceptive processes (physical reactions). The use of the UP strategies in six sessions was even found to successfully reduce loneliness during the COVID‐19 pandemic, even though this type of loneliness was not a specific treatment target.^43^ Interestingly, a session‐to‐session reduction of loneliness was more likely in participants who used a larger number of UP skills than they usually used on average.

Similar to the approach of the UP, the observed associations of loneliness with AMPD criteria A and B suggest that loneliness in PD can be effectively reduced by targeting various mechanisms on multiple levels, which may encompass perception, cognition, feelings, and behavior. In addition, interoceptive processes and physical reactions also play an important role and could be specifically addressed by body‐oriented techniques to alleviate loneliness.^44^, ^45^

Table 1 gives examples of strategies that may address feelings, thoughts, perceptual processes, physical reactions, and behavior associated with loneliness in PD. Theoretically, each strategy may specifically strengthen individual domains of personality functioning and reduce feelings of loneliness, although a valid holistic model of this interaction is still lacking.

**TABLE 1:** Examples of specific strategies to target loneliness in people with personality disorder that may warrant further research.

Domains	Examples for specific strategies
**Identity,** **self‐direction**	***Strategies for thinking*** ‐ Identify, evaluate, and reformulate negative appraisals of being alone ‐ Identify, evaluate, and reformulate automatic thoughts about oneself and others ‐ Identify, evaluate, and reformulate rejection expectations ‐ Identify, evaluate, and reformulate core beliefs ***Strategies for feeling*** ‐ Mindfully identify loneliness and associated emotions ‐ Recognize and acknowledge underlying needs ‐ Communicate needs if possible ‐ Otherwise, foster self‐compassion, self‐consolidation, and self‐connectedness ‐ Do the opposite to attenuate nonhelpful emotions ***Strategies for perception*** ‐ Identify social signs that trigger loneliness ‐ Practice interpersonal mindfulness by perceiving social signs mindfully and nonjudgmentally ‐ Recognize the impact of one's behavior on others ‐ Turn one's attention toward signs of positive social interaction‐Turn one's attention toward things one has in common with the person one is with ***Strategies to address adverse child experiences*** ‐ Gain insight into the interpersonal‐emotional history and its impact on the situation today (“loneliness biography”) ‐ Acknowledge needs of the past and practice self‐compassion and self‐consolidation
**Empathy,** **intimacy**	***Strategies for behavior*** ‐ Recognize the impact of dysfunctional behavior (e.g., avoidance behavior, aggressive or intrusive behavior, questioning of trust) and do the opposite in behavioral experiments ‐ Practice social skills (e.g., role play) ***Strategies to address social connectedness*** ‐ Clarify the value and function of relationships ‐ Formulate and pursue approach‐oriented social goals ‐ Find new relationships (e.g., via shared interests) ‐ Reactivate former relationships ‐ Talk about one's loneliness and feelings with others ‐ Take on responsibility for someone so that one feels one matters ‐ Ask for social support when needed ‐ Determine felt connectedness/loneliness with the therapist
	***Strategies for physical reactions*** ‐ Practice mindful perception of interoceptive sensations associated with loneliness ‐ Practice mindful breathing and relaxation exercises ‐ Calm oneself by self‐touch (e.g., tapping)

Strategies for thinking may be of specific importance because they address social cognition (i.e., negative core beliefs, negative appraisals, biased interpretations, and expectations) in both loneliness and PD. Furthermore, identifying and formulating approach‐oriented social goals may be beneficial.

Strategies for feeling encompass mindful awareness of loneliness‐associated emotions, such as shame,^12^ and specific needs. Acknowledging and satisfying needs (especially by communicating them and asking for social support or by self‐compassion) may alleviate loneliness, and practicing mindfulness and acceptance orientation in states of felt loneliness may ameliorate these states.^46^ In addition, nonhelpful accompanying emotions such as shame or guilt may be identified as nonhelpful and could be attenuated in the next step.

Strategies for perception may address social hypervigilance and perception biases for negative social cues (e.g., via interpersonal mindfulness training). In addition, awareness of one's own impact on others may be increased by the therapist's feedback on dysfunctional and rejection‐provoking behavior.

Turning to the putative roots of pervasive loneliness in PD, psychological interventions may be simultaneously guided by an individual's history of ACE. Both PD and loneliness are associated with an individual's recall of child maltreatment, especially emotional neglect and emotional abuse.^15^, ^16^ ACE and experiencing loneliness during childhood may predispose to feelings of loneliness later in life. Gaining insights into an individual's history of loneliness during childhood (e.g., by asking “Do you remember having felt alone as a child?”; “Did you play on your own and how do you remember such situations?”; “How did your significant others react when you felt lonely?”) may enable people with PD to identify core beliefs, fears, and expectations in relationships with significant others. Besides validating current feelings, cognitions, and behaviors associated with loneliness in terms of their historical antecedents, these experiences may also guide the therapist–client interaction, which could ideally lead to corrective relational experiences of a stable, reliable, containing, and caring relationship in therapy. Finally, a thorough assessment of ACE may help to explain an individual's impaired capacity for self‐compassion and self‐consolidation, both of which are decreased in lonely individuals with a history of ACE.^47^

Strategies for behavior encompass the recognition and change of maladaptive and dysfunctional behaviors associated with PD and loneliness (e.g., social avoidance, intrusive behavior, and constant questioning of trust). They include performing behavioral experiments and identifying, training, and shaping individually impaired social skills.

Loneliness factors related to the social environment can be addressed by focusing on enhancing social connectedness,^48^ increasing the size of one's social network, diversifying social contacts, asking for support, and strengthening the quality. It may be beneficial to clarify the desired quantity, functions, and quality of social relationships. Instead of having very few social contacts, such as one symbiotic but fragile relationship where rupture of the relationship may be particularly threatening, psychosocial interventions could aim at increasing the number and diversity of contacts in different areas of life including intimate partners, friends, family, and colleagues at work. Such interventions may work via social prescribing (e.g., by using social platforms/clubs and looking for shared interests). In addition, lonely individuals may be encouraged to strengthen the quality of relationships by talking about one's loneliness and emotions or increasing the emotional depth of relationships and to practice asking for emotional and/or social support. Interpersonal mindfulness may be helpful in this respect and has been operationalized as paying nonjudgmental and undivided attention to others and their interpersonal cues, instead of processing social information in a biased way.^49^ Moreover, interpersonal mindfulness has been suggested to moderate the association between loneliness and self‐compassion and to be linked to the meaning of life and has been shown to be a better predictor of interpersonal functioning than trait mindfulness.^50^ This mindful attention to moment‐by‐moment interactions between the self and others can attenuate a distorted perception of social cues and biased interpretations, both of which are associated with interpersonal problems in PD.

Finally, the physical reaction that can accompany loneliness may be targeted with mindfulness in the form of imagination and relaxation exercises or self‐calming exercises (e.g., by self‐touch). Mindfulness‐based stress reduction has been found to reduce loneliness in older adults and people with a variety of medical conditions.^51^

## CLINICAL IMPLICATIONS

Although studies in the past decade have identified common features of loneliness in relation to self‐ and interpersonal functioning, interindividual differences in these features are likely, also with respect to their etiopathogenesis and mechanisms of maintenance. Thus, establishing an individualized model of loneliness is essential for validating individual experiences and guiding therapy. Two clinical questions may be a good start for assessing loneliness in PD:

1. What specific features of loneliness are reported? This question is best answered by asking several subquestions: How is loneliness experienced in intimate and other relationships (including the relationship with the current or previous therapists)? How is this individual's loneliness related to current social connectedness and interactions, and what core patterns can be identified (i.e., specific perceptions of self and others, thoughts, associated feelings, physical reactions, and typical behavior)? How are these patterns anchored in impaired personality functioning, such as identity, self‐direction, empathy, and intimacy, and do specific dysfunctional traits (especially negative affectivity and detachment) contribute to loneliness? Are there specific experiences related to loss and grief or a history of ACE?
2. What are the factors maintaining this individual's loneliness from an intra‐ and/or interpersonal perspective? Examples of maintaining processes are self‐fulfilling rejection expectations, the fear of being vulnerable, a lack of social skills, and feelings of shame and guilt.

**FIGURE 1:**
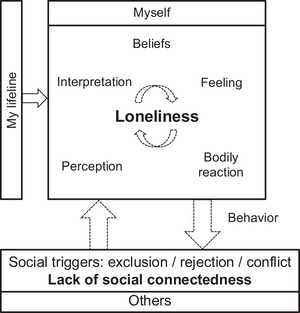
Model of loneliness in personality disorder for use with clients. Loneliness is accompanied by specific social perceptions, thoughts (interpretations and beliefs), feelings, bodily reactions, behaviors, and by reduced social connectedness, all of which may maintain loneliness (both intra‐ and interpersonally, dashed arrows) and represent intervention targets (see Table 1). Model adapted from Refs. 5 and 48.

**FIGURE 2:**
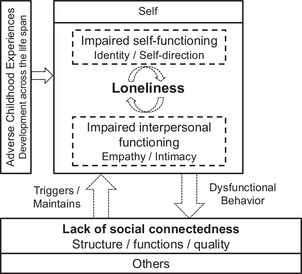
Model of loneliness in personality disorder for use by therapists that shows intra‐ and interpersonal maintaining cycles (dashed arrows). Loneliness is conceptualized as an unfulfilled need for connectedness that is predisposed by adverse childhood experiences and triggered by reduced social connectedness. It is accompanied by additional feelings, physical reactions, biased social perceptions (e.g., hypervigilance for rejection cues), and biased cognitions (e.g., rejection expectations) that may be anchored in impaired personality functioning. The resulting dysfunctional behavior may further decrease the quantity and quality of social relationships and reduce the availability of emotional/social support. Model adapted from Refs. 5 and 48.

For psychoeducation and validation in terms of individual characteristics and factors, the intra‐/interpersonal model of loneliness depicted in Figure 1 may be helpful for clients; additional components could be integrated as necessary. In addition, the therapist's view of this individualized model (Figure 2) may allow therapists to derive potential and personalized treatment strategies.

## DISCUSSION

Our call to action emphasizes the relevance of loneliness as a common experience with a strong impact on mental and physical health, especially in PD. Although mechanism‐based and effective psychotherapy models and psychological interventions are urgently needed for people living with PD, such approaches are still at an early stage of development. To combat loneliness, health care systems in various countries have laid the focus on the psychosocial environment (e.g., social prescribing in the UK, Canada, and other countries and Osekkai conferences in Japan). This approach is extremely important, but it may not adequately meet the particular needs of people with PD. Therefore, we call for the further development and investigation of PD loneliness interventions, which will need to specifically target impairments in the domains of self‐ and interpersonal functioning.^22^

Our model suggests using various psychological interventions to address loneliness and focuses on strategies that can be taught to clients during psychotherapy. However, loneliness in PD within treatment sessions may be additionally addressed by focusing on interpersonal aspects of the therapeutic relationship itself because this relationship has been identified as one of the most relevant factors for the effects of psychotherapy in PD.^52^ Several psychotherapy models, including transference‐focused psychotherapy (TFP),^53^ dialectical behavioral therapy (DBT),^54^ and the cognitive behavioral analysis system of psychotherapy (CBASP),^55^ explicitly focus on the therapeutic relationship and may, therefore, positively affect PD severity and loneliness. These three models are discussed below:

TFP, a psychodynamic treatment, was developed for the treatment of borderline PD and focuses specifically on the relationship (i.e., transference processes) between the patient and therapist. In TFP, which is rooted in object relations theory, loneliness can be conceptualized as related to deficits in cohesive and integrated mental representations of self and others. During normal development, an infant may develop the representation of a caring significant other, and this representation may become increasingly differentiated over time (e.g., a person may still be loving and caring even when they are currently absent). In contrast, lonely individuals may struggle with fragile or even polarized representations of others, which may lead, for instance, to increased fear of being alone or to highly ambivalent relationships with expectation biases toward rejection. These representations may become apparent within the patient‐therapist dyad via transference and can be clarified, interpreted, and integrated in therapy to enable the patient to develop a stable and cohesive perception of themself and others.

DBT is a cognitive behavioral treatment for individuals with borderline PD that focuses on emotion regulation (i.e., skills training) by combining and teaching various techniques in individual and group sessions. Besides suggesting a range of skills related to loneliness, such as self‐soothing, mindfulness, distraction, and checking the facts (i.e., contrasting loneliness with the fact of existing relationships), DBT also focuses on a reliable, genuine, and trustful therapeutic relationship. DBT therapists may share personal experiences with their clients (e.g., about their own experiences with loneliness) and are available even between therapy sessions via phone coaching. Phone coaching was designed to help a client to get through a crisis such as helping them to cope with a situation of intense loneliness. In addition, the mere knowledge of having an available and reliable therapist may help to combat loneliness.

CBASP also focuses on the therapeutic relationship and significantly reduces loneliness.^56^ Initially developed for people with chronic depression, CBASP was found to be effective independent of an additional comorbid PD^57^ and was superior to supportive psychotherapy in patients with a history of specific patterns of ACE.^58^ Several CBASP strategies (e.g., the technique of disciplined personal involvement with contingent personal responsivity and interpersonal discrimination exercises) may be used to address loneliness in PD^55^, ^56^. First, a feeling of loneliness within the therapeutic relationship may be transparently addressed by asking whether the individual feels lonely at the present moment with the therapist and how they think this feeling could be altered. Second, the therapist may provide feedback on dysfunctional behavior (e.g., hostility) within the therapeutic relationship that may be contributing to loneliness. Finally, the therapist may explicitly discriminate between the patient's expected and the therapist's actual reaction, like in a case of communicating needs or asking for support.

In the treatment of PD, integrated and personalized approaches have the potential to significantly alleviate loneliness and PD severity beyond the effects of established psychotherapy schools.^59^, ^60^ For instance, Gazzillo et al.^60^ stress the importance of pathogenic beliefs that people with PD have developed early in life because of ACE and that may be tested within the therapeutic relationship. By understanding a patient's expectations and resulting testing behavior (e.g., “Eventually, my therapist will abandon me”), treatment may be personalized to improve its outcome and offer corrective relational experiences to overcome loneliness within the therapeutic session.

Besides factors related to the therapeutic relationship, psychotherapy process research has identified several crucial factors for change in PD that are in line with the suggested strategies to reduce loneliness in PD. For example, Kramer et al.^52^ found that in PD, effective treatment is associated with emotional change (the improved regulation and awareness of emotion and needs); sociocognitive change (increased capacity to mentalize and recognize interpersonal patterns); and increased insight and self‐reflection.

## FUTURE RESEARCH

Future research is needed to test the effectiveness of the proposed strategies as stand‐alone interventions and in combination for treating loneliness in PD. This research needs to evaluate not only individual but also group therapies, which may be particularly promising for developing loneliness‐focused interventions because additional factors come into play. Yalom's primary factors in the therapeutic process would be applicable and they consist of the development of socializing techniques, imitative behavior, interpersonal learning, and group cohesion.^61^. Group therapies provide space to experience and foster social connections and may allow social learning. In addition, group settings that explicitly focus on social connectedness and loneliness may be superior to other formats, such as online‐based interventions. Similarly, a more intense day treatment setting may also foster social connectedness. However, the specific factors and requirements that effectively reduce loneliness still need to be identified.

In addition, further development of treatment modules for AMPD criteria A and B will allow us to analyze their effects on loneliness. Assessing the change in loneliness, personality functioning, and personality traits over time may be beneficial in the further analysis of the interplay of loneliness and PD. Starting this assessment early in life would enable researchers to take a developmental and longitudinal perspective, which may help in investigating the origins of both loneliness and PD in an individual's early interaction with the environment, especially in the context of ACE. Future research should prioritize the identification of the various pathways and mechanisms that link ACE and later loneliness and should use an interdisciplinary approach that brings together researchers from the field of pediatric, adolescent, and adult mental health care. In addition, experts with lived experience should be involved to help researchers to identify the most relevant research questions and clarify how to assess and differentiate individual variants of loneliness.

## CONCLUSIONS

Loneliness is a pervasive but understudied condition related to personality dysfunction in people living with PD, and individual loneliness may be related to deficits in domains of self‐ (i.e., identity and self‐direction) and interpersonal functioning (i.e., empathy and intimacy). Moreover, loneliness may contribute to both the severity and persistence of PD. Vice versa, specific difficulties of PD—dysfunctional core beliefs, impaired emotion regulation, and difficulties in developing and maintaining close and mutual relationships—may increase feelings of loneliness. Explicitly addressing loneliness in people with PD by using specific strategies on different levels and exploring the putative origin of loneliness in prior interpersonal experiences, including ACE, may be effective for ameliorating loneliness and reducing PD severity. Longitudinal studies of loneliness and the associated phenomena in PD are warranted across the lifespan to test current hypotheses on the etiopathogenesis of these conditions. In addition, clinical trials of mechanism‐based psychological interventions that target loneliness in people with PD are clearly needed and could inform researchers, clinicians, and health care providers about efficacious and cost‐effective interventions that could be combined with concepts of social prescribing and other current psychosocial programs.

## AUTHOR CONTRIBUTIONS

M.A.R.: Conceptualization, writing—original draft, writing—review and editing. F.P.: Conceptualization, writing—review and editing.

## CONFLICT OF INTEREST STATEMENT

F.P. is a member of the European Scientific Advisory Board of Brainsway Inc., Jerusalem, Israel, and the International Scientific Advisory Board of Sooma, Helsinki, Finland. He has received speakers’ honoraria from Mag&More GmbH and the neuroCare Group. His lab has received support with equipment from neuroConn GmbH, Ilmenau, Germany, and Mag&More GmbH and Brainsway Inc., Jerusalem, Israel. The remaining author declares that the research was conducted in the absence of any commercial or financial relationships that could be construed as a potential conflict of interest.

## PEER REVIEW

The peer review history for this article is available at: https://publons.com/publon/10.1111/nyas.15367.

## References

[1] Peplau, L. A. , & Perlman, D. (1982). Loneliness: A sourcebook of current theory, research and therapy. John Wiley & Sons Incorporated.

[2] Reichmann, F. F. (1959). Loneliness. Psychiatry, 22, 1–15. 13634274 10.1080/00332747.1959.11023153

[3] Ikhtabi, S. , Pitman, A. , Toh, G. , Birken, M. , Pearce, E. , & Johnson, S. (2022). The experience of loneliness among people with a “personality disorder” diagnosis or traits: A qualitative meta‐synthesis. BMC Psychiatry [Electronic Resource], 22, 1–17. 35177022 10.1186/s12888-022-03767-9 PMC8855579

[4] Ikhtabi, S. , Pitman, A. , Maconick, L. , Pearce, E. , Dale, O. , Rowe, S. , & Johnson, S. (2024). The prevalence and severity of loneliness and deficits in perceived social support among who have received a ‘personality disorder’ diagnosis or have relevant traits: A systematic review. BMC Psychiatry [Electronic Resource], 24, 21. 38172738 10.1186/s12888-023-05471-8 PMC10765693

[5] Reinhard, M. A. , Nenov‐Matt, T. , & Padberg, F. (2022). Loneliness in personality disorders. Current Psychiatry Reports, 24(11), 603‐612 . 10.1007/s11920-022-01368-7 PMC9525923 36181573

[6] Alasmawi, K. , Mann, F. , Lewis, G. , White, S. , Mezey, G. , & Lloyd‐Evans, B. (2020). To what extent does severity of loneliness vary among different mental health diagnostic groups: A cross‐sectional study. International Journal of Mental Health Nursing, 29, 921–934. 32356331 10.1111/inm.12727 PMC7616998

[7] Liebke, L. , Bungert, M. , Thome, J. , Hauschild, S. , Gescher, D. M. , Schmahl, C. , Bohus, M. , & Lis, S. (2017). Loneliness, social networks, and social functioning in borderline personality disorder. Personality Disorders, 8, 349–356. 27505189 10.1037/per0000208

[8] Nenov‐Matt, T. , Barton, B. B. , Dewald‐Kaufmann, J. , Goerigk, S. , Rek, S. , Zentz, K. , Musil, R. , Jobst, A. , Padberg, F. , & Reinhard, M. A. (2020). Loneliness, social isolation and their difference: A cross‐diagnostic study in persistent depressive disorder and borderline personality disorder. Frontiers in Psychiatry, 11, 608476. 33391058 10.3389/fpsyt.2020.608476 PMC7773662

[9] Pazzagli, A. , & Monti, M. R. (2000). Dysphoria and aloneness in borderline personality disorder. Psychopathology, 33, 220–226. 10867581 10.1159/000029147

[10] Kealy, D. , Woolgar, S. , & Hewitt, J. M. A. (2022). Investigating pathological narcissism and loneliness, and the link with life satisfaction. Scandinavian Journal of Psychology, 63, 32–38. 34524693 10.1111/sjop.12773

[11] Finch, E. F. , & Kealy, D. (2024). Loneliness in narcissistic vulnerability: Examining domains of personality functioning. Personality and Mental Health, 18, 259–268. 38666522 10.1002/pmh.1615

[12] Marčinko, D. , & Sutara, N. (2024). The role of psychodynamic and personality assessment in loneliness. Psychiatria Danubina, 36, 293–299. 39724116 10.24869/psyd.2024.293

[13] Ernst, M. , Brähler, E. , Kruse, J. , Kampling, H. , & Beutel, M. E. (2023). Does loneliness lie within? Personality functioning shapes loneliness and mental distress in a representative population sample. Journal of Affective Disorders Reports, 12, 100486.

[14] WHO. (2022). ICD‐11: International classification of diseases (11th revision) . 10.1097/YCO.0000000000000656 33252429

[15] de Heer, C. , Bi, S. , Finkenauer, C. , Alink, L. , & Maes, M. (2022). The association between child maltreatment and loneliness across the lifespan: A systematic review and multilevel meta‐analysis. Child Maltreatment , 29(2), 388–404. 10.1177/10775595221103420 PMC11539460 35652822

[16] Reinhard, M. A. , Rek, S. V. , Nenov‐Matt, T. , Barton, B. B. , Dewald‐Kaufmann, J. , Merz, K. , Musil, R. , Jobst, A. , Brakemeier, E. L. , Bertsch, K. , & Padberg, F. (2022). Association of loneliness and social network size in adulthood with childhood maltreatment: Analyses of a population‐based and a clinical sample. European Psychiatry, 65(1), e55 . 10.1192/j.eurpsy.2022.2313 PMC9491078 36059118

[17] Sabaß, L. , Buchenrieder, N. , Rek, S. V. , Nenov‐Matt, T. , Lange, J. , Barton, B. B. , Musil, R. , Jobst, A. , Padberg, F. , & Reinhard, M. A. (2022). Attachment mediates the link between childhood maltreatment and loneliness in persistent depressive disorder. Journal of Affective Disorders, 312, 61–68. 35728677 10.1016/j.jad.2022.06.021

[18] Heleniak, C. , Jenness, J. L. , Vander Stoep, A. , Mccauley, E. , & Mclaughlin, K. A. (2016). Childhood maltreatment exposure and disruptions in emotion regulation: A transdiagnostic pathway to adolescent internalizing and externalizing psychopathology. Cognitive Therapy and Research, 40, 394–415. 27695145 10.1007/s10608-015-9735-z PMC5042349

[19] Dagan, Y. , & Yager, J. (2019). Addressing loneliness in complex PTSD. Journal of Nervous and Mental Disease, 207, 433–439. 31045977 10.1097/NMD.0000000000000992

[20] Holt‐Lunstad, J. , Smith, T. B. , Baker, M. , Harris, T. , & Stephenson, D. (2015). Loneliness and social isolation as risk factors for mortality: A meta‐analytic review. Perspectives on Psychological Science, 10, 227–237. 25910392 10.1177/1745691614568352

[21] Hickin, N. , Käll, A. , Shafran, R. , Sutcliffe, S. , Manzotti, G. , & Langan, D. (2021). The effectiveness of psychological interventions for loneliness: A systematic review and meta‐analysis. Clinical Psychology Review, 88, 102066. 34339939 10.1016/j.cpr.2021.102066

[22] Akhter‐Khan, S. C. , & Au, R. (2020). Why loneliness interventions are unsuccessful: A call for precision health. Advances in Geriatric Medicine and Research , 2(3), e200016. 10.20900/agmr20200016 PMC9410567 36037052

[23] Masi, C. M. , Chen, H. Y. , Hawkley, L. C. , & Cacioppo, J. T. (2011). A meta‐analysis of interventions to reduce loneliness. Personality and Social Psychology Review, 15, 219–266. 20716644 10.1177/1088868310377394 PMC3865701

[24] Welch, V. , Ghogomu, E. T. , Dowling, S. , Barbeau, V. I. , Al‐Zubaidi, A. A. A. , Beveridge, E. , Bondok, M. , Desai, P. , Doyle, R. , Huang, J. , Hussain, T. , Jearvis, A. , Jahel, F. , Madani, L. , Choo, W. Y. , Yunus, R. M. , Tengku Mohd, T. A. M. , Wadhwani, A. , Ameer, A. A. , … Mikton, C. (2024). In‐person interventions to reduce social isolation and loneliness: An evidence and gap map. Campbell Systematic Reviews, 20, e1408.

[25] Spithoven, A. W. M. , Bijttebier, P. , & Goossens, L. (2017). It is all in their mind: A review on information processing bias in lonely individuals. Clinical Psychology Review, 58, 97–114. 29102150 10.1016/j.cpr.2017.10.003

[26] Riddleston, L. , Bangura, E. , Gibson, O. , Qualter, P. , & Lau, J. Y. F. (2023). Developing an interpretation bias modification training task for alleviating loneliness in young people. Behaviour Research and Therapy, 168, 104380. 37541156 10.1016/j.brat.2023.104380

[27] Käll, A. , Shafran, R. , Lindegaard, T. , Bennett, S. , Cooper, Z. , Coughtrey, A. , & Andersson, G. (2020). A common elements approach to the development of a modular cognitive behavioral theory for chronic loneliness. Journal of Consulting and Clinical Psychology, 88, 269–282. 32068427 10.1037/ccp0000454

[28] APA. (2013). Diagnostic and statistical manual of mental disorders (DSM‐5®). American Psychiatric Publication. 10.1590/s2317-17822013000200017 24413388

[29] Kunz, J. I. , Frey, A. , Bertsch, K. , Barton, B. B. , Blei, L. , Schirle, H. M. , Konvalin, F. , Jobst, A. , Musil, R. , Padberg, F. , & Reinhard, M. A. (2023). Loneliness is associated with lower self‐ and clinician‐rated levels of personality functioning. Journal of Personality Disorders, 37, 724–740. 38038658 10.1521/pedi.2023.37.6.724

[30] Spitzer, C. , Müller, S. , Kerber, A. , Hutsebaut, J. , Brähler, E. , & Zimmermann, J. (2021). Die deutsche Version der Level of Personality Functioning Scale‐Brief Form 2.0 (LPFS‐BF): Faktorenstruktur, konvergente Validität und Normwerte in der Allgemeinbevölkerung. PPmP‐Psychotherapie Psychosomatik Medizinische Psychologie, 71, 284–293. 10.1055/a-1343-2396 33694153

[31] Hutsebaut, J. , Berghuis, H. , De Saeger, H. , Kaasenbrood, A., & Ingenhoven, T. (2014). Semi‐structured interview for personality functioning DSM‐5 (STiP 5.1). Trimbos Institute.

[32] Labonté, L. E. , & Kealy, D. (2023). Understanding loneliness: The roles of self‐ and interpersonal dysfunction and early parental indifference. Bulletin of the Menninger Clinic, 87, 266–290. 37695883 10.1521/bumc.2023.87.3.266

[33] Ehrenthal, J. C. , Kruse, J. , Schmalbach, B. , Dinger, U. , Werner, S. , Schauenburg, H. , Brähler, E. , & Kampling, H. (2023). Measuring personality functioning with the 12‐item version of the OPD‐Structure Questionnaire (OPD‐SQS): Reliability, factor structure, validity, and measurement invariance in the general population. Frontiers in Psychology, 14, 1248992. 37780157 10.3389/fpsyg.2023.1248992 PMC10536238

[34] Gamache, D. , Savard, C. , Leclerc, P. , & Côté, A. (2019). Introducing a short self‐report for the assessment of DSM–5 level of personality functioning for personality disorders: The Self and Interpersonal Functioning Scale. Personality Disorders: Theory, Research, and Treatment, 10, 438–447. 10.1037/per0000335 31033325

[35] Hayward, D. , MacIntyre, D. , & Steele, D. (2024). Borderline personality disorder is an innate empathy anomaly: A scoping and narrative review. International Journal of Psychiatry in Clinical Practice, 28, 152–166. 39470631 10.1080/13651501.2024.2420662

[36] Romero, E. , & Alonso, C. (2019). Maladaptative personality traits in adolescence: Behavioural, emotional and motivational correlates of the PID‐5‐BF scales. Psicothema, 3, 263–270. 10.7334/psicothema2019.86 31292040

[37] Roche, M. J. , Pincus, A. L. , & Cole, P. E. (2019). Linking dimensions and dynamics in psychopathology research: An example using DSM‐5 instruments. Journal of Research in Personality, 82, 103852.

[38] Freilich, C. D. , Mcgue, M. , South, S. C. , Roisman, G. I. , & Krueger, R. F. (2024). Connecting loneliness with pathological personality traits: Evidence for genetic and environmental mediation from a study of older twins. Personality Disorders, 15, 34–45. 37498698 10.1037/per0000635 PMC11166192

[39] Chau, A. K. C. , So, S. H. , Sun, X. , Zhu, C. , Chiu, C.‐D. , Chan, R. C. K. , & Leung, P. W.‐L. (2022). A network analysis on the relationship between loneliness and schizotypy. Journal of Affective Disorders, 311, 148–156. 35594977 10.1016/j.jad.2022.05.057

[40] Widiger, T. A. , & Hines, A. (2022). The Diagnostic and Statistical Manual of Mental Disorders, Fifth Edition alternative model of personality disorder. Personality Disorders, 13, 347–355. 35787119 10.1037/per0000524

[41] Barlow, D. H. , Ellard, K. K. , & Fairholme, C. P. (2010). Unified protocol for transdiagnostic treatment of emotional disorders: Workbook. Oxford University Press. 10.1016/j.cbpra.2009.06.002 PMC7986982 33762811

[42] Ellard, K. K. , Fairholme, C. P. , Boisseau, C. L. , Farchione, T. J. , & Barlow, D. H. (2010). Unified protocol for the transdiagnostic treatment of emotional disorders: Protocol development and initial outcome data. Cognitive and Behavioral Practice, 17, 88–101. 33762811 10.1016/j.cbpra.2009.06.002 PMC7986982

[43] Southward, M. W. , Terrill, D. R. , & Sauer‐Zavala, S. (2022). The effects of the Unified Protocol and Unified Protocol skills on loneliness in the COVID‐19 pandemic. Depression and Anxiety, 39, 913–921. 36372958 10.1002/da.23297 PMC9877890

[44] Arnold, A. J. , Winkielman, P. , & Dobkins, K. (2019). Interoception and social connection. Frontiers in Psychology, 10, 2589. 31849741 10.3389/fpsyg.2019.02589 PMC6901918

[45] Quadt, L. , Esposito, G. , Critchley, H. D. , & Garfinkel, S. N. (2020). Brain−body interactions underlying the association of loneliness with mental and physical health. Neuroscience and Biobehavioral Reviews, 116, 283–300. 32610178 10.1016/j.neubiorev.2020.06.015

[46] Lindsay, E. K. , Young, S. , Brown, K. W. , Smyth, J. M. , & Creswell, J. D (2019). Mindfulness training reduces loneliness and increases social contact in a randomized controlled trial. Proceedings of the National Academy of Sciences, 116, 3488–3493. 10.1073/pnas.1813588116 PMC6397548 30808743

[47] Qu, Y. (2024). From childhood emotional abuse to adolescent loneliness: The roles of self‐compassion and rejection sensitivity. Child Abuse & Neglect, 156, 107020. 39236348 10.1016/j.chiabu.2024.107020

[48] Holt‐Lunstad, J. (2021). The major health implications of social connection. Current Directions in Psychological Science, 30, 251–259.

[49] Suh, H. , & Lee, J. H. (2023). Linking loneliness and meaning in life: Roles of self‐compassion and interpersonal mindfulness. International Journal of Applied Positive Psychology, 8, 365–381.

[50] Pratscher, S. D. , Rose, A. J. , Markovitz, L. , & Bettencourt, A. (2018). Interpersonal mindfulness: Investigating mindfulness in interpersonal interactions, co‐rumination, and friendship quality. Mindfulness, 9, 1206–1215.

[51] Saban, K. L. , Collins, E. G. , Mathews, H. L. , Bryant, F. B. , Tell, D. , Gonzalez, B. , Bhoopalam, S. , Chroniak, C. P. , & Janusek, L. W. (2022). Impact of a mindfulness‐based stress reduction program on psychological well‐being, cortisol, and inflammation in women veterans. Journal of General Internal Medicine, 37, 751–761. 36042095 10.1007/s11606-022-07584-4 PMC9481828

[52] Kramer, U. , Beuchat, H. , Grandjean, L. , & Pascual‐Leone, A. (2020). How personality disorders change in psychotherapy: A concise review of process. Current Psychiatry Reports, 22, 1–9. 32519017 10.1007/s11920-020-01162-3

[53] Clarkin, J. F. , Yeomans, F. E. , & Kernberg, O. F. (1999). Psychotherapy for borderline personality. John Wiley & Sons Inc.

[54] Linehan, M. M. (1993). Skills training manual for treating borderline personality disorder. Guilford Press.

[55] McCullough, J. P., Jr. (2003). Treatment for chronic depression using Cognitive Behavioral Analysis System of Psychotherapy (CBASP). Journal of Clinical Psychology, 59, 833–846. 12858425 10.1002/jclp.10176

[56] Reinhard, M. A. , Zentz, K. , Nenov‐Matt, T. , Barton, B. B. , Rek, S. V. , Goerigk, S. , Brakemeier, E. L. , Musil, R. , Jobst, A. , & Padberg, F. (2021). Cognitive Behavioral Analysis System of Psychotherapy reduces loneliness in patients with persistent depressive disorder. Journal of Affective Disorders Reports, 5, 100171.

[57] Erkens, N. , Schramm, E. , Kriston, L. , Hautzinger, M. , Härter, M. , Schweiger, U. , & Klein, J. P. (2018). Association of comorbid personality disorders with clinical characteristics and outcome in a randomized controlled trial comparing two psychotherapies for early‐onset persistent depressive disorder. Journal of Affective Disorders, 229, 262–268. 29329058 10.1016/j.jad.2017.12.091

[58] Goerigk, S. , Elsaesser, M. , Reinhard, M. A. , Kriston, L. , Härter, M. , Hautzinger, M. , Klein, J. P. , McCullough, J. P. , Schramm, E. , & Padberg, F. (2024). Childhood Trauma Questionnaire‐based child maltreatment profiles to predict efficacy of the Cognitive Behavioral Analysis System of Psychotherapy versus non‐specific psychotherapy in adults with early‐onset chronic depression: Cluster analysis of data from a randomised controlled trial. Lancet Psychiatry, 11, 709–719. 39147459 10.1016/S2215-0366(24)00209-8

[59] Clarkin, J. F. , Cain, N. , & Livesley, W. J. (2015). An integrated approach to treatment of patients with personality disorders. Journal of Psychotherapy Integration, 25, 3–12.

[60] Gazzillo, F. , Dazzi, N. , Kealy, D. , & Cuomo, R. (2021). Personalizing psychotherapy for personality disorders: Perspectives from control‐mastery theory. Psychoanalytic Psychology, 38, 266–278.

[61] Yalom, I. D. , & Leszcz, M. (2020). The theory and practice of group psychotherapy. Basic Books.

